# A case report: percutaneous management of high-output heart failure from iatrogenic aortocoronary venous grafting to the coronary sinus

**DOI:** 10.1186/s43044-021-00186-1

**Published:** 2021-07-05

**Authors:** Akarsh Parekh, Vivek Sengupta, Ryan Malek, Mark Zainea

**Affiliations:** 1 Department of Cardiovascular Medicine, McLaren Macomb-Oakland Medical Center, 1000 Harrington Street, Mount Clemens, MI 48043 USA; 2 Michigan State University School of Medicine, East Lansing, MI USA; 3 Wayne State University School of Medicine, Detroit, MI USA

**Keywords:** Aortocoronary fistula, Arteriovenous fistula, Iatrogenic vascular fistula, Percutaneous fistula coiling, High-output heart failure, Coronary artery disease

## Abstract

**Background:**

Aortocoronary arteriovenous fistula (ACAVF) due to iatrogenic bypass grafting to a cardiac vein is an exceedingly rare complication resulting from coronary artery bypass grafting (CABG) surgery. If not identified in a timely fashion, ACAVF has known significant clinical consequences related to left to right shunting and possible residual myocardial ischemia.

**Case presentation:**

An 82-year-old male with a history of CABG, presented with dyspnea. Over the span of 2 years following CABG, the patient experienced progressive exertional dyspnea and peripheral edema. The patient was found to have a new cardiomyopathy with a severely reduced ejection fraction at 30–35%. The patient underwent diagnostic left heart catheterization, and an ACAVF was discovered between a saphenous vein graft and the coronary sinus. The patient underwent successful percutaneous coiling of the ACAVF with no residual flow. Follow-up echocardiography at 3 months revealed restoration of left ventricular systolic function to 50% and significant improvement in heart failure symptoms.

**Conclusions:**

ACAVF is an exceedingly rare iatrogenic complication of CABG that may result in residual ischemia from the non-grafted myocardial territory and other sequelae relating to left to right shunting and a high-output state. Management for this pathology includes but is not limited to the use of percutaneous coiling, implantation of covered stents, graft removal and regrafting, and ligation.

**Supplementary Information:**

The online version contains supplementary material available at 10.1186/s43044-021-00186-1.

## Background

In 1951, Vineberg first reported the use of the internal mammary artery to revascularize the cardiac muscle by directly implanting it to the myocardium [1]. In 1958, the first coronary artery bypass grafting (CABG) with internal mammary artery was performed by Longmire [1]. Typical complications from a CABG included stroke, vein graft occlusion, acute myocardial infarction, angina, damage to the aorta, bleeding, and death [2]. Iatrogenic aortocoronary arteriovenous fistula (ACAVF) due to unintentional grafting of a vessel to a coronary sinus is an extremely rare complication of CABG [2–4]. ACAVF patients can present with angina, dyspnea, fatigue, ventricular arrhythmia, or heart failure [1]. These patients need to be monitored for symptoms or new-onset continuous murmurs [1]. Most patients experience symptoms between 6 weeks and 4 years after their CABG [1]. Symptom presentation can be delayed especially in patients with small ACAVF [1].

ACAVF can go undiagnosed in patients who are asymptomatic or have significantly less clinical manifestations [3]. ACAVF can lead to significant morbidity [3]. ACAVF can lead to left to right shunting, precipitating high-output cardiac failure [3]. A left to right shunt can cause increased intracardiac pressures on the right side of the heart and a step up in oxygen saturations [2]. Patients with untreated hemodynamically significant ACAVF eventually can develop symptoms and complications [2]. Consequently, if the shunting is untreated, it can predispose to bacterial endocarditis, severe systemic to pulmonary shunting, or fistula rupture [3, 4]. In the long term, arterialization of the coronary vein or sinus can lead to its enlargement and cause external compression of the left circumflex artery [2]. There are several risk factors that can predispose to iatrogenic grafting to a coronary vein or sinus. These factors include scarring and fibrosis from a past pericardial disease, previous history of CABG, myocardial infarction, or presence of epicardial fat that can lead an operator to have difficulty in localizing the distribution of a coronary artery [3, 5].

In asymptomatic patients with ACAVF, medical management and observation, without any intervention, is recommended [3]. Deligonul et al. reported two cases whose asymptomatic iatrogenic AVACF spontaneously closed [3]. However, in patients with symptoms or those refractory to medical therapy, a closure of ACAVF and grafting of the diseased coronary artery via repeat CABG or fistula ligation would be required [3, 5]. Percutaneous catheter interventions can eradicate the need for thoracotomy, decrease recovery time, and reduce hospital stay and cost with better safety profile compared to surgical options [1]. Most of the cases of iatrogenic ACAVF are found with the use of saphenous vein graft compared to internal mammary artery [1].

## Case presentation

An 82-year-old male, with a history of CABG, who presented to an outpatient clinic with dyspnea on exertion and angina. He has a past medical history of coronary artery disease, hypertension, diabetes mellitus type 2, hyperlipidemia, peripheral vascular disease, permanent atrial fibrillation, chronic obstructive pulmonary disease, and Meniere disease. His home medications include aspirin, coumadin, tamsulosin, vitamin D3, carvedilol, digoxin, insulin glargine, insulin aspart, finasteride, fluticasone, sennosides, torsemide, albuterol inhaler, tiotropium inhaler, budesonide/formoterol inhaler, and atorvastatin. He is allergic to lisinopril. The patient quit smoking 35 years ago and occasionally drinks alcohol. He underwent a four-vessel CABG 14 months ago, wherein the left internal mammary artery was grafted to his left anterior descending artery; saphenous vein was grafted to his obtuse marginal, diagonal branch and right coronary artery. He subsequently developed post-operative atrial fibrillation during the initial hospitalization for CABG. Initially, his dyspnea was attributed to his atrial fibrillation. His proposed symptomatic atrial fibrillation was refractory to pharmacologic rate and rhythm control. He subsequently underwent electrical cardioversion to alleviate his symptoms with restoration of sinus rhythm, within 3 months of patient developing atrial fibrillation. However, he continued to have progressive exertional dyspnea with peripheral edema despite optimal medical therapy and diuresis.

**Fig. 1:**
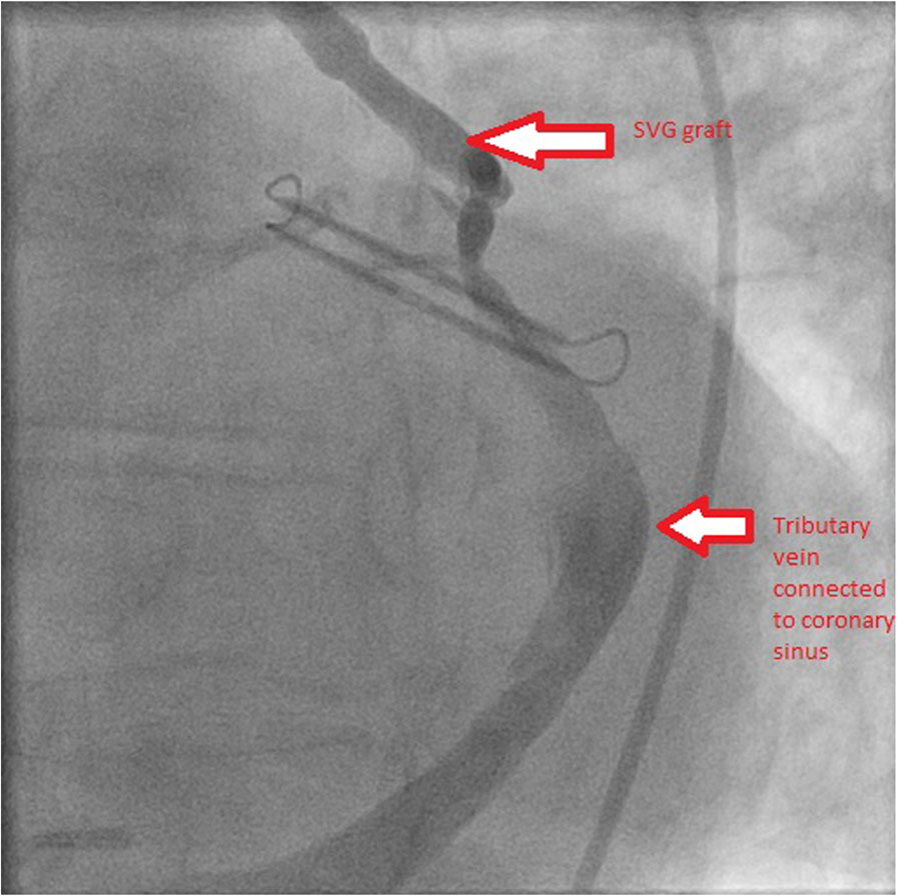
Fistula between saphenous vein graft anastomosed to a tributary vein connected to the coronary sinus (arrows)


An echocardiogram was performed which showed a new cardiomyopathy with a severely reduced left ventricular ejection fraction of 30–35%. Multiple echocardiograms following his CABG had consistently demonstrated preserved left ventricular systolic function. Further evaluation of his new cardiomyopathy was performed using a vasodilator nuclear stress test, which was negative. Due to persistent symptoms, a coronary angiography was performed. Coronary angiography demonstrated a saphenous venous graft that was anastomosed to a tributary vein with retrograde filling of the coronary sinus, instead of the obtuse marginal branch of the left circumflex artery (Fig. 1, Video 1). The intracardiac left to right shunting was hemodynamically significant with Qp/Qs of 1.8, leading to pulmonary hypertension and subsequent right ventricular dilatation. The patient had a pulmonary artery pressure of 62/27 with a mean of 45 mmHg.

**Additional file 1: Video 1**: Fistula between saphenous vein graft anastomosed to a tributary vein connected to the coronary sinus.

**Fig. 2:**
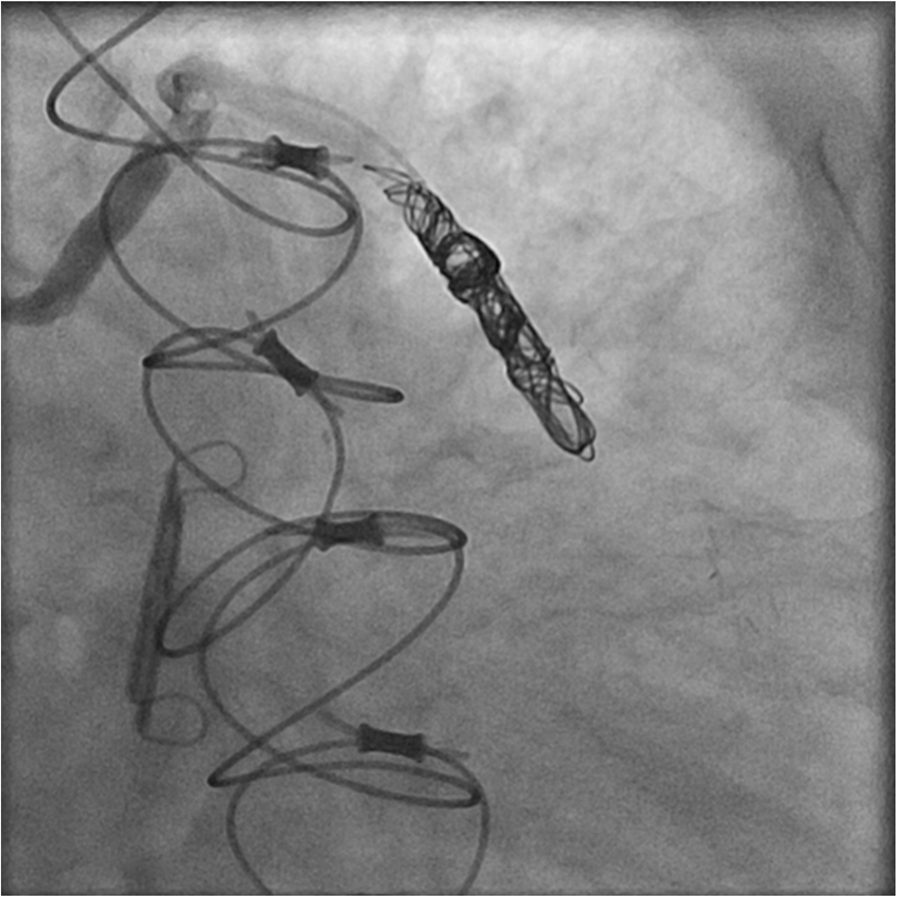
An angiographic image of successful coiling of the aortocoronary fistula


Using a multidisciplinary approach and collaboration with interventional radiology, the decision was made to pursue a percutaneous approach for ACAVF closure. The procedure was performed using a 2.4 French microcatheter which was advanced over a 0.014-inch microwire into the mid/distal aspect of the saphenous venous graft. Four detachable 0.018-inch microcoils were placed into the graft until it was completely occluded (Video 2). A follow-up angiogram confirmed the position and almost complete resolution of the ACAVF (Fig. 2, Video 3). No contrast was visualized going into the coronary sinus. Follow-up echocardiography, performed 3 months post-coiling, revealed restoration of left ventricular systolic function to 50% and significant improvement in heart failure symptoms.

**Additional file 2: Video 2**: An angiogram showing coiling of the aortocoronary fistula


**Additional file 3: Video 3**: An angiogram that displays almost complete resolution of the aortocoronary fistula

## Conclusion

An acquired ACAVF can be seen as a complication from cardiac surgery, myocardial infarction, endomyocardial biopsy, pacemaker implantation, coronary angiography, or chest trauma [2]. In our patient, a retrospective review of diagnostic testing over 2 years following CABG revealed gradual worsening of pulmonary hypertension and right ventricular dimensions by echocardiographic analysis (Table 1). These changes due to left to right shunting resolved following the percutaneous coiling. Utilization of percutaneous coiling technique to occlude flow through the vein graft to cardiac vein fistula effectively eliminated left to right shunting and improved symptomatic burden, and subsequent echocardiography showed significant improvement of right ventricular systolic pressure and right heart dimensions. A multidisciplinary approach and collaborating with interventional radiology in our case was effective in identifying the pathology and selecting the optimal management plan for successfully closing ACAVF.

**Table 1:**
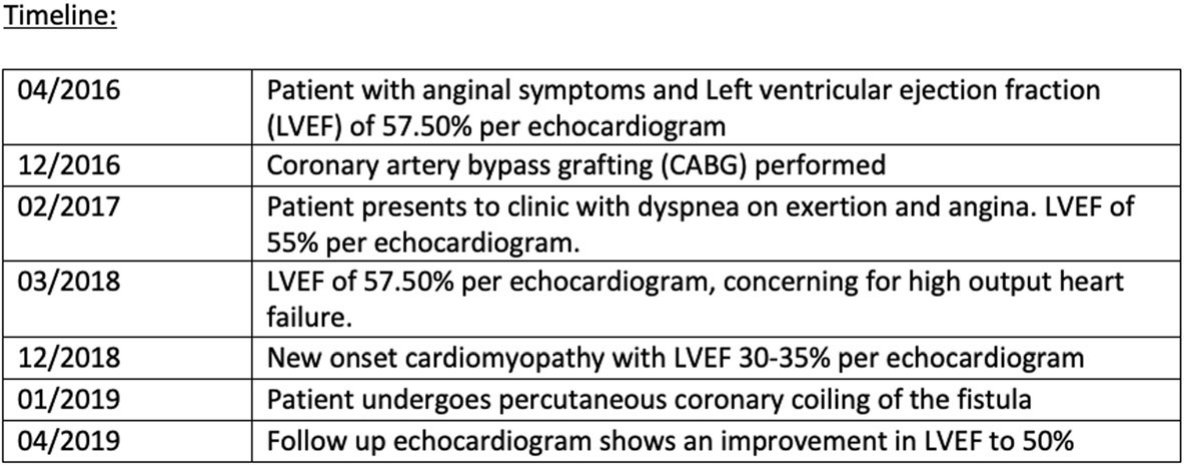
Timeline of patient’s clinical and diagnostic history

## Data Availability

N/A

## Abbreviations

CABG: Coronary artery bypass grafting
ACAVF: Aortocoronary arteriovenous fistula

## References

[1] Jung IS, Jeong JO, Kim SS, Shin BS, Shin SK, Park YK, Jin SA, Ahn KT, Seong IW (2011). Iatrogenic left internal mammary artery to great cardiac vein anastomosis treated with coil embolization. Korean Circ J..

[2] Wayangankar SA, Saucedo JF (2015). Transcatheter coiling of saphenous vein graft to coronary sinus after coronary artery bypass surgery: a case report. J Cardiovasc Med (Hagerstown).

[3] Gardner JD, Maddox WR, JB C (2012). Iatrogenic aortocoronary arteriovenous fistula following coronary artery bypass surgery: a case report and complete review of the literature. Case Rep Cardiol.

[4] Ornek E, Kundi H, Kiziltunc E, Cetin M (2016). Treatment of iatrogenic aortocoronary arteriovenous fistula coronary covered stent. Case Rep Cardiol..

[5] Sheiban I, Moretti C, Colangelo S (2006). Iatrogenic left internal mammary artery–coronary vein anastomosis treated with covered stent deployment via retrograde percutaneous coronary sinus approach. Catheter Cardiovasc Interv..

